# Microbiome analysis reveals the intestinal microbiota characteristics and potential impact of *Procambarus clarkii*

**DOI:** 10.1007/s00253-023-12914-5

**Published:** 2024-01-10

**Authors:** Ming Xu, Fulong Li, Xiaoli Zhang, Baipeng Chen, Yi Geng, Ping Ouyang, Defang Chen, Liangyu Li, Xiaoli Huang

**Affiliations:** 1 Department of Aquaculture, College of Animal Science & Technology, Sichuan Agricultural University, Chengdu, 611130 Sichuan China; 2 Fishery Research Institute, Chengdu Academy of Agriculture and Forestry Sciences, Wenjiang, Sichuan China; 3 Department of Basic Veterinary Medicine, College of Veterinary Medicine, Sichuan Agricultural University, Chengdu, 611130 Sichuan China

**Keywords:** Crayfish, High-throughput sequencing, Intestinal microbiota, Digestion, qRT-PCR

## Abstract

**Abstract:**

The intestinal microbiota interacts with the host and plays an important role in the immune response, digestive physiology, and regulation of body functions. In addition, it is also well documented that the intestinal microbiota of aquatic animals are closely related to their growth rate. However, whether it resulted in different sizes of crayfish in the rice-crayfish coculture model remained vague. Here, we analyzed the intestinal microbiota characteristics of crayfish of three sizes in the same typical rice-crayfish coculture field by high-throughput sequencing technology combined with quantitative real-time polymerase chain reaction (qRT-PCR) and enzyme activity, investigating the relationship between intestinal microbiota in crayfish and water and sediments. The results showed that the dominant intestinal microbiota of crayfish was significantly different between the large size group (BS), normal size group (NS), and small size group (SS), where *Bacteroides* and *Candidatus_Bacilloplasma* contributed to the growth of crayfish by facilitating food digestion through cellulolysis, which might be one of the potential factors affecting the difference in sizes. Follow-up experiments confirmed that the activity of lipase (LPS) and protease was higher in BS, and the relative expression of development-related genes, including alpha-amylase (*α-AMY*), myocyte-specific enhancer factor 2*a* (*MEF2a*), glutathione reductase (*GR*), chitinase (*CHI*), and ecdysone receptor (*EcR*), in BS was significantly higher than that in SS. These findings revealed the intestinal microbiota characteristics of crayfish of different sizes and their potential impact on growth, which is valuable for managing and manipulating the intestinal microbiota in crayfish to achieve high productivity in practice.

**Key points:**

• *Significant differences in the dominant microflora of BS, NS, and SS in crayfish.*

• *Cellulolysis might be a potential factor affecting different sizes in crayfish.*

• *Adding Bacteroides and Candidatus_Bacilloplasma helped the growth of crayfish.*

**Supplementary Information:**

The online version contains supplementary material available at 10.1007/s00253-023-12914-5.

## Introduction

The crayfish (*Procambarus clarkii*) is a member of the Cambaridae family, which is popular with consumers because of its delicious meat and has become an economically important aquaculture species in recent years (Feng et al. 2021). As a dominant species in natural environments, crayfish has the characteristics of strong adaptability and fast growth and usually has two breeding periods in 1 year (Shu 2014). In 2022, crayfish production reached 2.89 million tons, an increase of 9.76%, accounting for 8.79% of total freshwater aquaculture production in China (China Fisheries Association 2023).

With consumer markets expanded and culture models updated, rice-crayfish coculture production has become the dominant model (Si et al. 2017; Xu et al. 2022). Compared with traditional pond culture, the model of rice-crayfish coculture utilized the leisure production period of paddies, greatly improved the utilization ratio, and reduced the inputs of fertilizers and pesticides, which not only improved the productivity of rice-crayfish coculture fields but also facilitated the growth of crayfish, resulting in significant economic benefits to farmers (Hou et al. 2021; Kruse et al. 2020). Therefore, these advantages greatly contributed to the widespread use of rice-crayfish coculture, accounting for 61.85% of the total production of crayfish in China (Association 2023). However, the value of crayfish varied greatly by size. The price data from the Chinese market in 2022 (data from market survey) showed that large crayfish (weighing more than 22 g) could achieve 20–30 RMB/500 g and small crayfish (less than 18 g) could achieve only 10–15 RMB/500 g, a difference of approximately 2.62 million RMB depending on the output of 1 t. Thus, farmers prefer to acquire larger individuals, generating more income. However, what caused this difference is still unknown.

A number of studies have found that the intestinal microbiota can significantly affect the growth of mammal (Beaumont et al. 2022; Wu et al. 2023). Similarly, the intestinal microbiota is also closely associated with the growth of aquatic animals. For example, the intestinal microbiota could enhance the growth of hosts by degrading polysaccharides and promoting vitamin synthesis and also affect the growth rate by improving or reducing the digestion and absorption of nutrients (Fan et al. 2019), resulting in differences in morphology (Fassarella et al. 2021; Garibay-Valdez et al. 2020; Zhang et al. 2023). Crayfish, as benthic organisms located between water and sediments, are greatly influenced by the microbiota of the environment, whose intestinal microbiota are interdependent and constrain each other (Fan and Li 2019). In addition, the digestion and absorption of nutrients from their hosts would improve or decrease when the dominant bacteria changed (Wang et al. 2020a). Therefore, there is reason for suspicion that the different sizes of crayfish might be associated with their intestinal microbiota even when living in the same paddy fields, which is worth studying.

In summary, to explore the difference in the intestinal microbiota in different sizes of crayfish and the relationship with water and sediments, we collected water, sediment, and intestine samples from a typical rice-crayfish coculture field in Xinjin, China, a suitable climate with preference for rice-crayfish coculture, and analyzed the intestinal microbiota characteristics of crayfish in three sizes and the relationship of water and sediments by high-throughput sequencing technology combined with quantitative real-time polymerase chain reaction and digestive enzyme activity assays, which attempted to provide informative suggestions for culture engineering decision-makers.

## Materials and methods

### Crayfish and rearing conditions

**Fig. 1:**
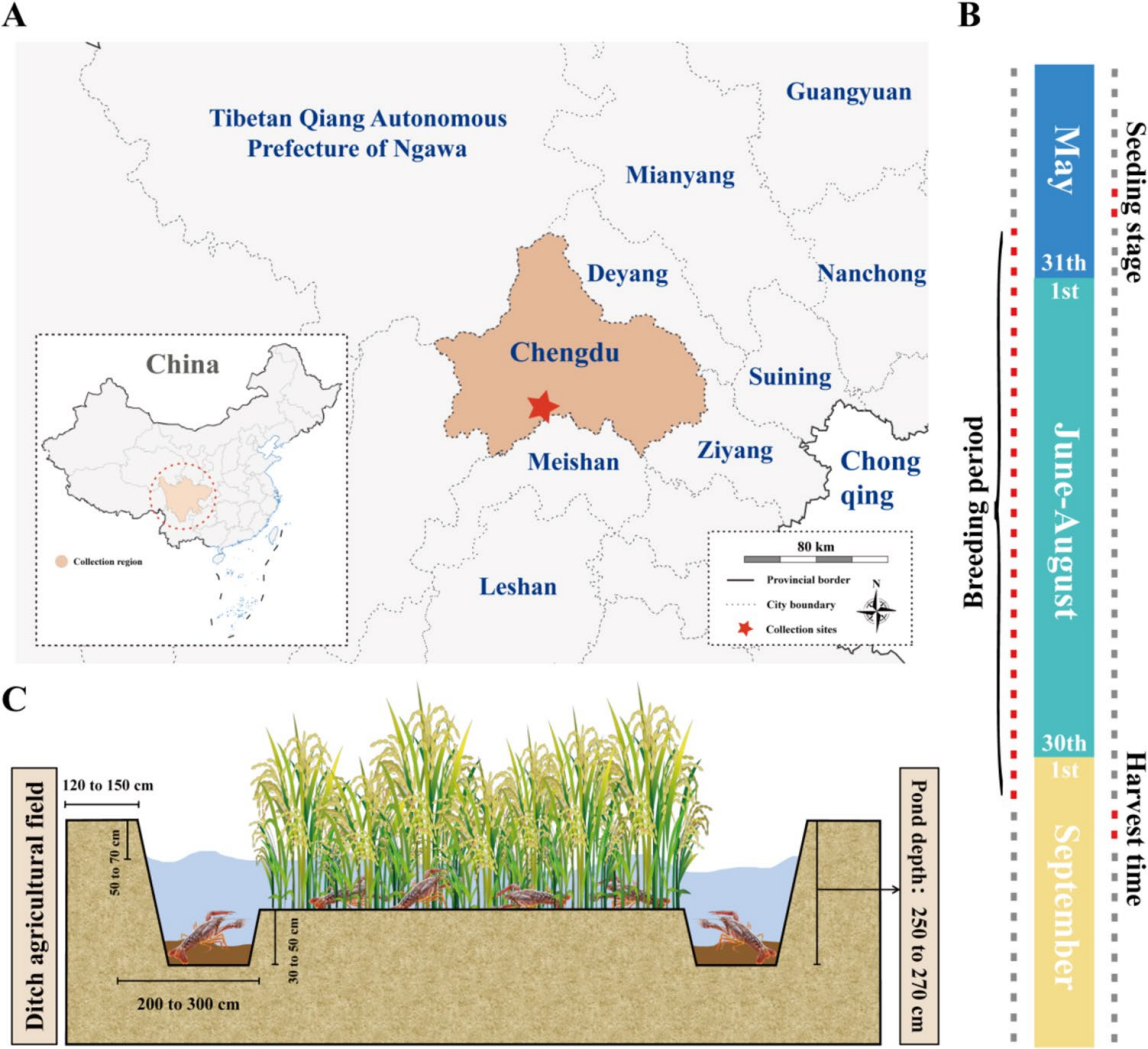
Basic information for sample collection. **A** Geographical distribution of the collection region. ★ shows the collection site in Xinjin, Chengdu, Sichuan Province, China (103° 76′ E, 30° 22′ N). **B** The timeline of data collection. **C** Schematic diagram of the rice-crawfish coculture field

The site of the experiment was selected in Xinjin District, Sichuan Province, China (103° 76′ E, 30° 22′ N), covering an area of over 8000 m^2^, located in a typical rice-crayfish coculture region in the western part of the Chengdu Plain (Fig. 1A). The climate was subtropical monsoonal humid, with an aggregate annual rainfall of 987 mm and an average temperature of 16.4 °C, which is suitable for rice-crayfish coculture. The rice-crayfish coculture region was completely and thoroughly cleaned and disinfected by the researchers before the beginning of the experiment for successful execution. The rice had been planted before the crayfish were put in. The seedlings produced from the same batch of breeder crayfish were selected for input in May 2022 to avoid age differences when they were of similar size (1.96 ± 0.11 g), and the density was approximately 80 individuals/m^2^. During the rearing process, the same commercial feed (Tongwei, Chengdu, China) was fed at 3% of the total weight twice daily (7:00–8:00 and 17:00–18:00), and the breeding logs for the study area from May 2022 to September 2022 were provided by the technicians (Fig. 1B). A schematic diagram of the rice-crawfish coculture region is shown in Fig. 1C.

### Sampling of water quality and weather data

Water quality and weather data during the sampling period (September 25, 2022) were noted. The water parameters included water depth, temperature, pH, dissolved oxygen, ammonia nitrogen, nitrite, and transparency. The water depth was defined as the distance from the bottom of the ditch to the water surface, measured by the five-point method. The water temperature was determined below 20 cm of the water surface by a thermometer (CONJANT, Shanghai, China). The dissolved oxygen, pH, ammonia nitrogen, and nitrite in water were measured by a portable water quality tester (HACH, Shanghai, China). The transparency of water was assessed by Behcet’s disk method (Huang et al. 2022). All indicators were repeated three times to minimize errors. Meanwhile, the meteorology logs for the study area were provided by the website (http://www.hjhj-e.com/). All data were recorded in Excel (Microsoft, Redmond, WA, USA) and counted.

### Sample collection

The typical rice-crayfish coculture field with 4 months of crayfish culturing in the present year was selected for sampling, including water, sediment, and intestine of crayfish, on September 25, 2022. The crayfish stopped rearing 1 day before sampling. A total of ninety crayfish, which were randomly collected at the same time, sampled from this field were divided into three groups, a large size group (BS, body weight ≥ 22 g/individual), a normal size group (NS, 18 g < body weight < 22 g per individual), and a small size group (SS, body weight ≤ 18 g/individual), based on body weight and market price. During sampling, the dorsal carapace of crayfish was opened, and the whole intestine was cut off and rinsed three times with sterile phosphate buffer. Three crayfish intestines in the same group were combined into one sample for 16S rRNA gene amplification and miSeq sequencing, qRT-PCR validation, and enzyme activity analysis, and three duplicate samples in one group were collected to minimize errors in the outdoor data. Water samples were collected at 50 cm depth from the surface of five different sites and mixed, and then, a 500-mL water mixture was selected and filtered through a 0.22-µm microfiltration membrane as one microbial sample. Five hundred gram sediment samples were collected through the same method. The water and sediment samples were also repeated three times. All samples were rapidly frozen in liquid nitrogen and stored in a − 80 °C refrigerator for preservation until use. The corresponding label information of the samples is shown in Supplemental Table S1.

### DNA extraction, bacterial 16S rRNA gene amplification, and miSeq sequencing

The total genomic DNA of the microbial community was extracted according to the manufacturer’s instructions (Omega, Norcross, GA, USA), the quality of the extracted genomic DNA was measured by agarose gel electrophoresis at 1%, and the concentration and purity of DNA were determined using a NanoDrop 2000 (Thermo Scientific, Fresno, CA, USA). The extracted DNA was used as a template, and the upstream primer 338F (5′-ACTCCTACGGGAGGCAGCAG-3′) carrying the barcode sequence and the downstream primer 806R (5′-GGACTACHVGGGTWTCTAAT-3′) (Schloss et al. 2009) were used for PCR amplification of the V3-V4 variable region of the 16S rRNA gene. Three replicates were performed for each sample. The PCR products were detected on 2% agarose gels, purified using the AxyPrep DNA Gel Extraction Kit (Axy Biosciences, Union City, CA, USA), and quantified using a Quantus™ fluorometer (Promega, Madison, WI, USA). High-throughput sequencing was performed using the Illumina MiSeq PE 300 platform (Illumina, San Diego, CA, USA).

### Bioinformatics analysis

All data analysis was performed on the Megabio Cloud platform (https://cloud.majorbio.com) as follows: Using QIIME (version 1.9.1, http://qiime.org/install/index.html) to remove ineligible reads from the raw data and passing through the SILVA rRNA database, utilizing Uparse (version 7.0.1090, http://www.drive5.com/uparse/), the filtered sequences were clustered into operational taxonomic units (OTUs) with a 70% confidence level. OTUs were annotated at a 97% similarity level by using an RDP classifier (version 2.13, https://sourceforge.net/projects/) to acquire OTU annotation information. Based on the annotation information, the bacterial community composition was analyzed at the phylum and genus levels. Alpha diversity (Sobs index, Ace index, Shannon index, Simpson index, and Chao index) was calculated using Mothur software (version 1.30.2, http://www.mothur.org/wiki/Calculators) (Wang et al. 2020a), and the Wilcoxon rank sum test was used for the intergroup differences. Beta-diversity with arithmetic mean (UPGMA) and hierarchical clustering trees were used to compare the similarity of the composition of the comparative clusters (Segata et al. 2011). The phylum and genus were selected for correlation network graph analysis (NetworkX (version 1.11, https://networkx.org)) based on Spearman correlation |*r|*> 0.6 *P* < 0.05 (Barberán et al. 2012). Functional abundance profiles of bacterial communities in different systems were inferred and obtained from 16S rRNA marker gene sequences using PICRUSt II (version 1.10, http://picrust.github.io/picrust/). Network analysis (NetworkX (version 1.11, https://networkx.org)) was used to represent the distribution between samples and phylum and genus levels and to calculate genus-to-genus correlations. The artificially constructed FAPROTAX database (Louca et al. 2016) was used to annotate the functions of biological taxa.

### Analysis of enzyme activity

The intestinal samples were thawed and cut longitudinally along the intestinal wall, weighed, then thoroughly homogenized in 10 volumes (w/v) of ice-cold physiological saline solution and centrifuged at 2500 × g at 4 °C for 10 min. The resultant supernatants were collected and stored at − 20 °C for enzymatic determinations. In this experiment, the activities of the intestine digestive enzyme (protease and lipase) were determined using a protease assay kit (No. A080-2–1) and lipase assay kit (No. A054-1–1), respectively. The above kits were all produced by Nanjing Jiancheng Institute of Biological Engineering (Jiancheng Bioengineering Ltd., Nanjing, China). One unit of protease activity was defined as the amount of hydrolysis of casein that liberated 1 μg of tyrosine per minute under the conditions of the assay (pH 8.0 and 37 °C). One unit of lipase activity was defined as the quantity of enzyme that liberated 1 μmol of butyric acid per minute under the conditions of the assay (pH 7.0 and 37 °C).

### Quantitative real-time PCR analysis

To further verify different gene expression levels for growth, digestion, and absorption in crayfish, five relative genes were randomly selected for qRT-PCR analysis. The specific steps were as follows: total RNA was isolated from the intestine by using an RNAiso Plus Kit (Takara, Dalian, China) according to the manufacturer’s instructions followed by DNase I treatment. The quality and quantity were assessed by agarose gel electrophoresis at 1% and spectrophotometric analysis at 260 nm and 280 nm. Subsequently, the total RNA was reverse transcribed into cDNA by using a Prime Script™ RT reagent Kit (Takara, Dalian, China) according to the manufacturer’s instructions. A quantitative real-time PCR detection system (Bio-Rad Laboratories, Inc., Hercules, CA, USA) using a SYBR® PrimeScript RT-PCR Kit II (Takara, Dalian, China) was performed for the genes for glutathione reductase (*GR*), myocyte-specific enhancer factor 2*a* (*MEF2a*), alpha-amylase (*α-AMY*), ecdysone receptor (*EcR*), and chitinase (*CHI*) and a housekeeping gene (*β-actin*) according to standard protocols with the primer sequences indicated in Table 1. Briefly, quantitative real-time PCR was performed in a total volume of 10 μL containing 5 μL of TB Green™ Premix Ex Taq™ II, 0.2 μL of Rox, 1 μL of cDNA, 0.8 μL of each primer, and 3 μL of double-distilled water. The reaction conditions used were as follows: 95 °C for 3 min, followed by 39 cycles of 95 °C for 10 s, 54.6 °C for 30 s, and 72 °C for 30 s, with the dissolution curve increasing from 0.5 to 95 °C every 5 s. All qRT-PCRs were performed in triplicate, and target specificity was determined based on dissociation curve analysis. *β-Actin* was selected as the internal control to normalize the expression level of each gene. The relative expression level of the target gene versus the *β-actin* gene was calculated using the 2^−ΔΔCT^ method (Liu et al. 2023, 2022; Sun et al. 2020).

**Table 1:** Primers for qRT-PCR of crayfish intestine

Gene	Primer sequence (5′-3′)	Size (bp)	GenBank accession number
*GR*	F: CTGGAGTACGGTTGTTGTGG	95	XM_045739602.1
	R: AAGGGTTGGTAGTAGGAATGG		
*MEF2a*	F: CATCTTCCAACCATCCTGGG	126	XM_045740704.1
	R: GTTTGCTCAACGGGGTATCA		
*α-AMY*	F: CTCCGACAACCTCGACTTCC	126	XM_045758626.1
	R: TAGCAGTTCCGCACGTTGTA		
*EcR*	F: TTCCCGAGTCTCAATGCC	366	XM_045758116.1
	R: GAAGAGTGCCGAAACCAG		
*CHI*	F: TTTGACTCGGTGGGTGCT	385	XM_045766906.1
	R: TGTATGGTCCAGGCTTTCC		
*β-Actin*	F: TATCCTGCGTCTGGACTTGG	108	XM_045725833.1
	R: CGAACGATTTCTCGCTCTGC		

*F* forward primer, *R* reverse primer, *GR* gene for glutathione reductases *MEF2a* gene for myocyte-specific enhancer factor 2*a*, *α-AMY* gene for alpha-amylase, *EcR* gene for ecdysone receptor, *CHI* gene for chitinase, *β-actin* gene for beta-actin

### Database statistics

All data are expressed as the mean ± SD deviation. Significant differences were determined using Student’s *t* test and nonparametric Wilcoxon signed-rank tests with statistical significance levels accepted by *P* < 0.05. Statistical analysis and data visualization were performed on the Megabio Cloud platform (https://cloud.majorbio.com).

## Results

### Site characteristics

Supplemental Table S2 and Supplemental Table S3 show the water quality and weather data of the sampling site. The results showed that the water quality parameters and weather conditions were suitable for crayfish growth. Of all crayfish cultured in the same field, BS, NS, and SS accounted for 35%, 40%, and 25% of the total production, respectively. Based on random sampling and grouping results, the weight of crayfish in BS was 25.98 ± 1.08 g, while that in NS and SS was only 19.32 ± 0.65 g and 14.84 ± 1.39 g, respectively, showing significant differences.

### Global analysis of 16S rDNA sequencing

To explore the reasons for the difference in crayfish sizes, as well as the potential association with the environment, we collected three sizes of crayfish intestines (BS, NS, SS), water, and sediments for global analysis of 16S rDNA sequencing (Supplemental Table S1). After quality filtering and assignment, a total of 748,842 high-quality sequences with an average length of 422 bp were obtained (Supplemental Table S1). The Shannon index was used to calculate species diversity, which showed that bacterial diversity was highest in sediments, intermediate in the water, and lowest in the intestine. For crayfish, the highest diversity was in BS and the lowest in SS (Supplemental Table S4). The Sobs and Ace indices used to calculate community richness showed a similar trend to the diversity analysis (Supplemental Table S5). In addition, the rank abundance and core also illustrated the differences in community richness among different types of samples (Supplemental Fig. S1). Good’s coverage of each sample, which estimated the completeness of sequencing, was > 99.88%, demonstrating that the sequence identified reflected the preponderance of bacteria in each sample (Supplemental Table S1).

### Species annotation and assessment reveal the differences in samples

A total of 929 OTUs were annotated, belonging to 443 genera of 33 phyla. The intestine samples showed a similar annotation, while the water and sediment samples included large numbers of unique species (Supplemental Fig. S2A). The proportion of dominant species composition and distribution of each sample was reflected by Circos plots, which showed that *Candidatus_Bacilloplasma* was the most dominant bacterium in intestine sample, followed by *norank_f__norank_o_RsaHf231* and *Bacteroides* (Supplemental Fig. S3). Similarly, differences in bacterial community composition of intestines in crayfish and water and sediments were visualized by principal component analysis (PCA), which suggested that crayfish in BS and SS showed a different distribution tendencies, and NS was between the other two groups. Meanwhile, the water and sediments were distant from the intestine, indicating a greater difference (Supplemental Fig. S2B).

### Significant differences in dominant bacteria of the intestine in crayfish

**Fig. 2:**
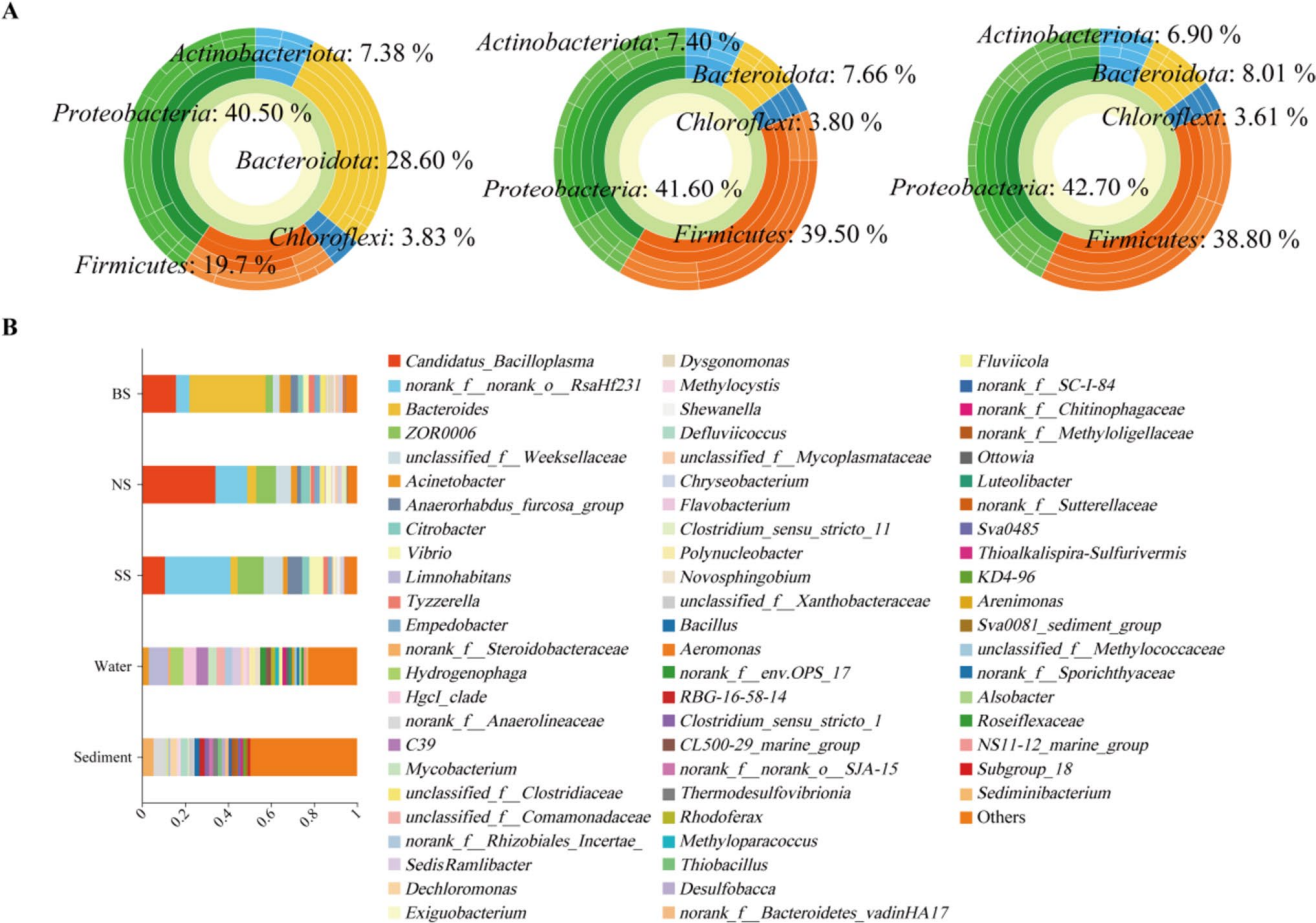
Structure of the bacterial community in the intestine, water, and sediments. **A** Multilevel species sunburst diagram, annotated at the phylum level for intestine samples of three sizes in crayfish. **B** Classification analysis of intestine, water, and sediment samples at the genus level by column plots

Using species compositional analysis, we explored the difference in dominant bacteria in all samples. The different types of samples showed similar annotations, while the abundance varied. The multilevel species sunburst diagram (Fig. 2A) showed that the bacterial composition of the intestine belonged to five main phyla, i.e., Firmicutes (mean abundance = 19.7% in BS, 39.5% in NS and 38.8% in SS), Bacteroidota (mean abundance = 28.6% in BS, 7.66% in NS, 8.01% in SS), and Proteobacteria (mean abundance = 40.5% in BS, 41.6% in NS, 42.7% in SS). The composition of the remaining two phyla was similar. The pie charts showed the annotation of the bacterial community at the phylum level for the water and sediment samples, where Proteobacteria were predominantly dominant (Supplemental Fig. S4). Among them, the abundance of Firmicutes in BS was significantly lower than that of others, while Bacteroidota was significantly more abundant, which seemed that some differences existed with the previous studies (Sun et al. 2023a,b; Wang et al. 2021). In contrast, the differences in Proteobacteria were not significant, which could be caused by the same environment, with no differences in the intestine. In addition, when comparing the bacteria of the intestine with water and sediments, the differences in genus levels are presented by bar charts (Fig. 2B). The predominant bacterial genus annotated in water and sediments showed a noticeable increase, being a significant difference from the crayfish intestine. Overall, these results indicated that there were remarkable variations in the dominant bacterial genera among different sizes of intestine, and *Bacteroides* was the most dominant in BS. The sample hierarchical clustering is shown in Supplemental Fig. S5.

### The intestinal microbiota of crayfish in SS was more susceptible to environmental influences

**Fig. 3:**
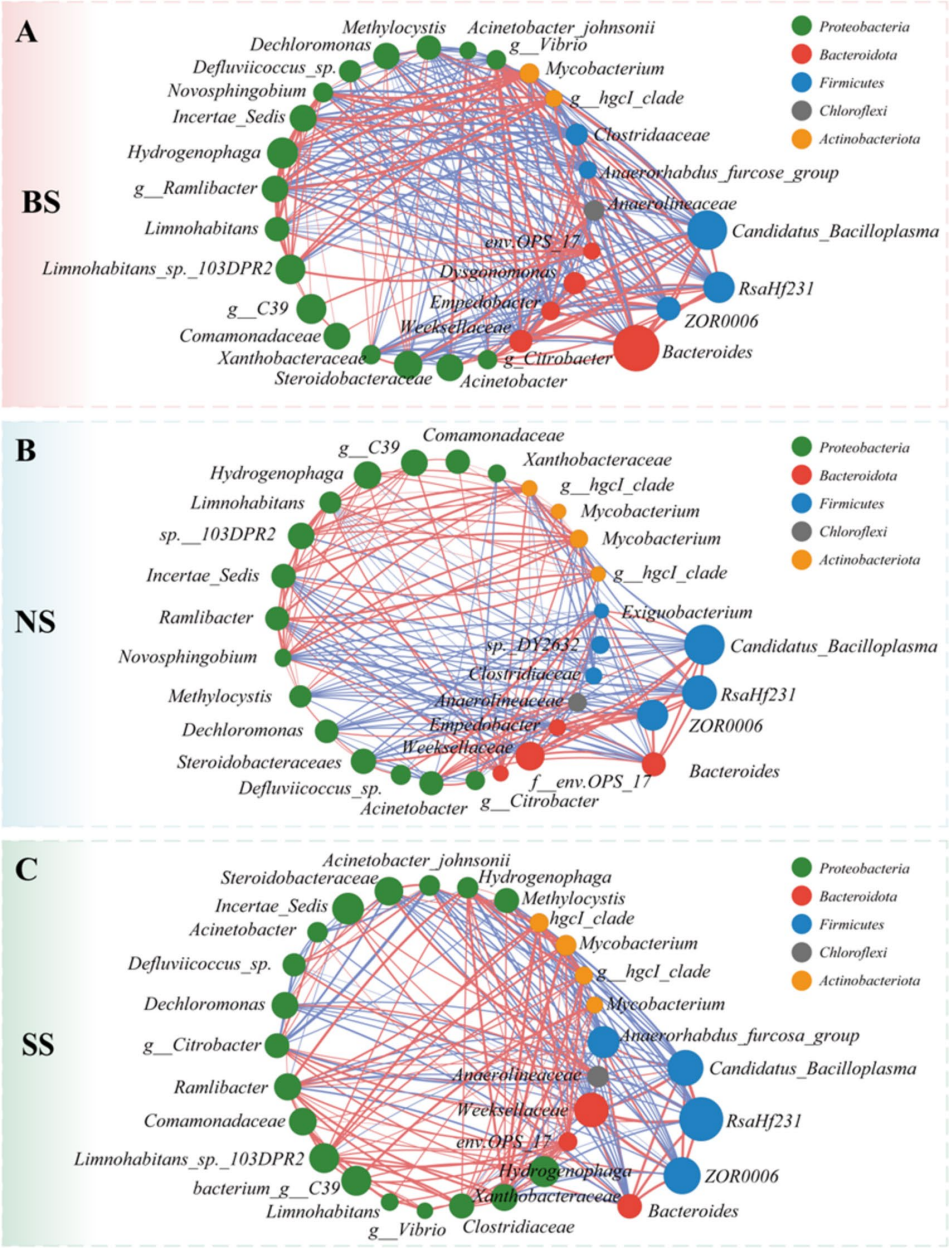
Correlation networks of bacterial community structures of water, sediment and intestine. **A**–**C** Correlation network in BS (**A**), NS (**B**), and SS (**C**). The size of the dots indicates the abundance of the 30 most abundant OTUs. The size of the nodes in the figure indicates the species abundance size, and the red and blue lines indicate positive and negative correlations between the connecting points

Despite the differences in intestinal microbiota between the three sizes of crayfish, the relationship with the environment is worth exploring. Interestingly, Chloroflexi, Firmicutes, Actinobacteria, Bacteroidetes, and Proteobacteria were shared, and another twenty-four phyla were unique to water and sediments. In addition, Bacteroides, Limnohabitans, Dysgonomonas, and Steroidobacteraceae were the dominant phyla (Supplemental Fig. S6 and Supplemental Table S5). We further sought to explore potential interactions between different intestine samples. Correlation networks were used to screen for dominant species in the total community, determining the importance of species in the network based on degree, closeness, and betweenness centrality values. The results showed that *Candidatus_Bacilloplasma*, *Bacteroides*, and *ZOR0006* were the dominant species in BS, while *norank_f__norank_o_RsaHf231* was dominant in SS (Fig. 3A, C and Supplemental Table S6, S8). There was a bidirectional influence in NS, being regulated by multiple bacteria (Fig. 3B and Supplemental Table S7).

### Functional predictions revealed that digestion and absorption were more dominant in large crayfish

**Fig. 4:**
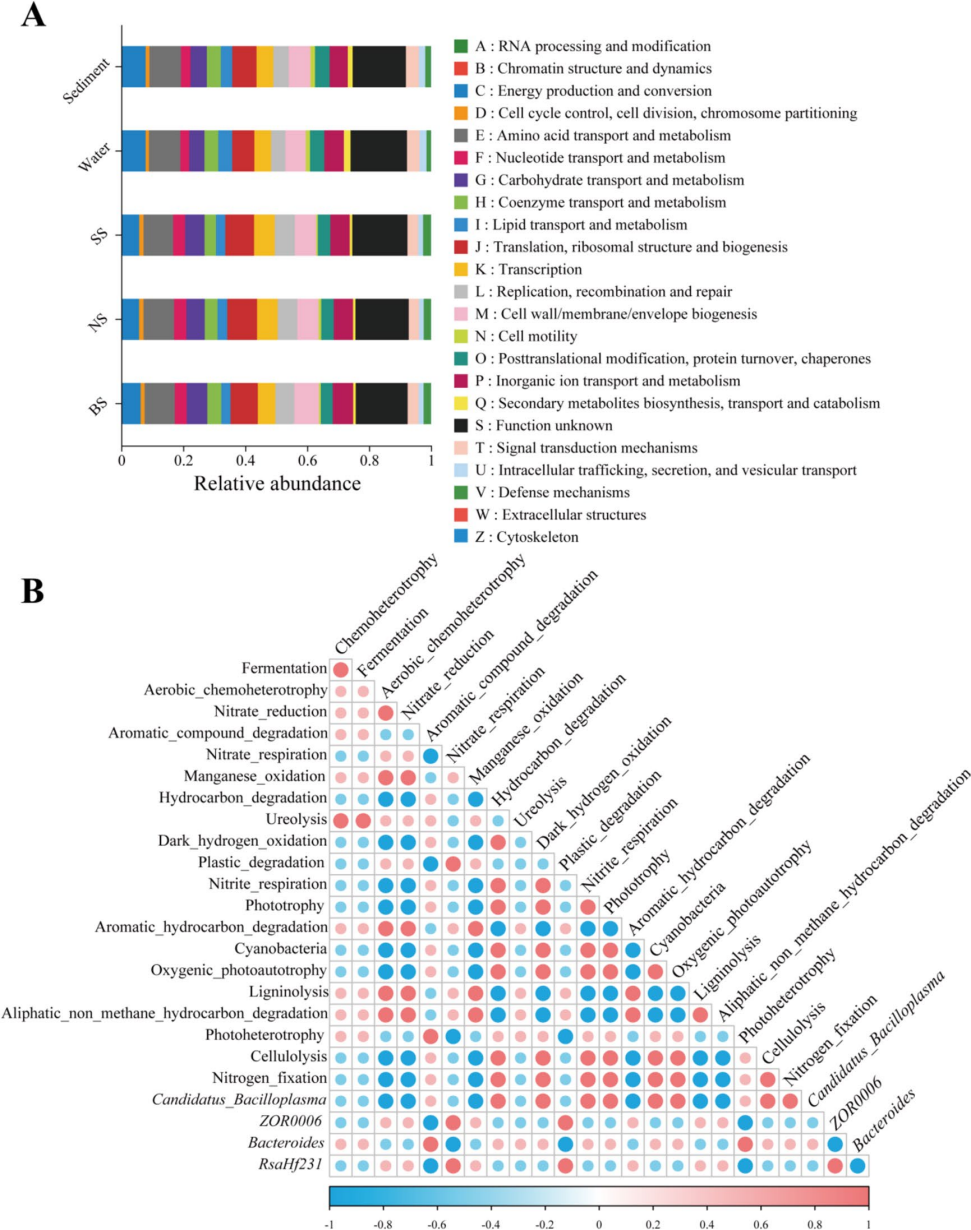
Functional prediction of microbial communities in all samples. **A** COG prediction of the intestine, water, and sediments. **B** Prediction of FAPROTAX functions in dominant and critical bacteria. The circle indicates the magnitude of the correlation, with red and blue showing positive and negative correlations, respectively

High-throughput sequencing analysis showed that the intestinal microbiota of crayfish was correlated with the environment and that those were more consistent in BS. We would further like to explain the reasons for the differences; however, there were no significant differences by Clusters of Orthologous Groups of proteins (COG) prediction (Fig. 4A). Similarly, using the FAPROTAX database predicted the digestion and absorption function of intestinal bacterial communities based on individual differences. The results showed that there were more annotated cellulolysis, nitrogen fixation, aromatic compound degradation, and nitrate ammonification pathways in BS. In contrast, “nitrite_denitrification” and “plastic_degradation” were more pronounced in SS, which was not beneficial for the digestion and absorption of crayfish. (Fig. 4B).

### Intestinal digestive enzyme activity

**Fig. 5:**
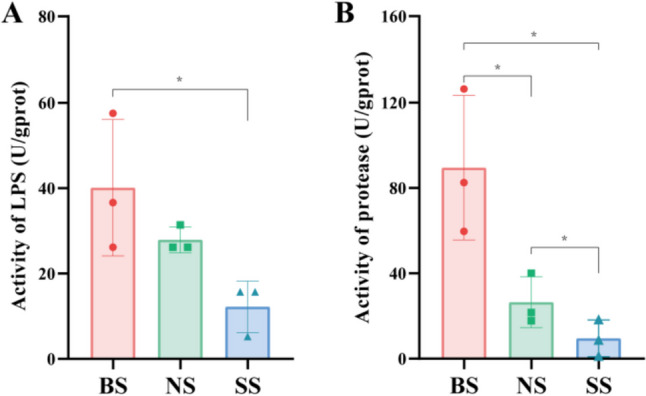
The intestinal digestive enzymes of BS, NS, and SS in crayfish. **A** The activity of lipase (LPS). **B** The activity of protease. Significance: *P* < 0.05 (*) (*n* = 3)

Figure 5 shows that the activities of intestinal digestive enzymes were significantly different. The LPS and protease activities were both significantly higher in BS than in SS (*P* < 0.05). The lipase activity of NS was not significantly different from that of BS and SS (*P* > 0.05), while the protease activity was different (*P* < 0.05).

### The expression levels of genes varied significantly in different sizes of crayfish

**Fig. 6:**
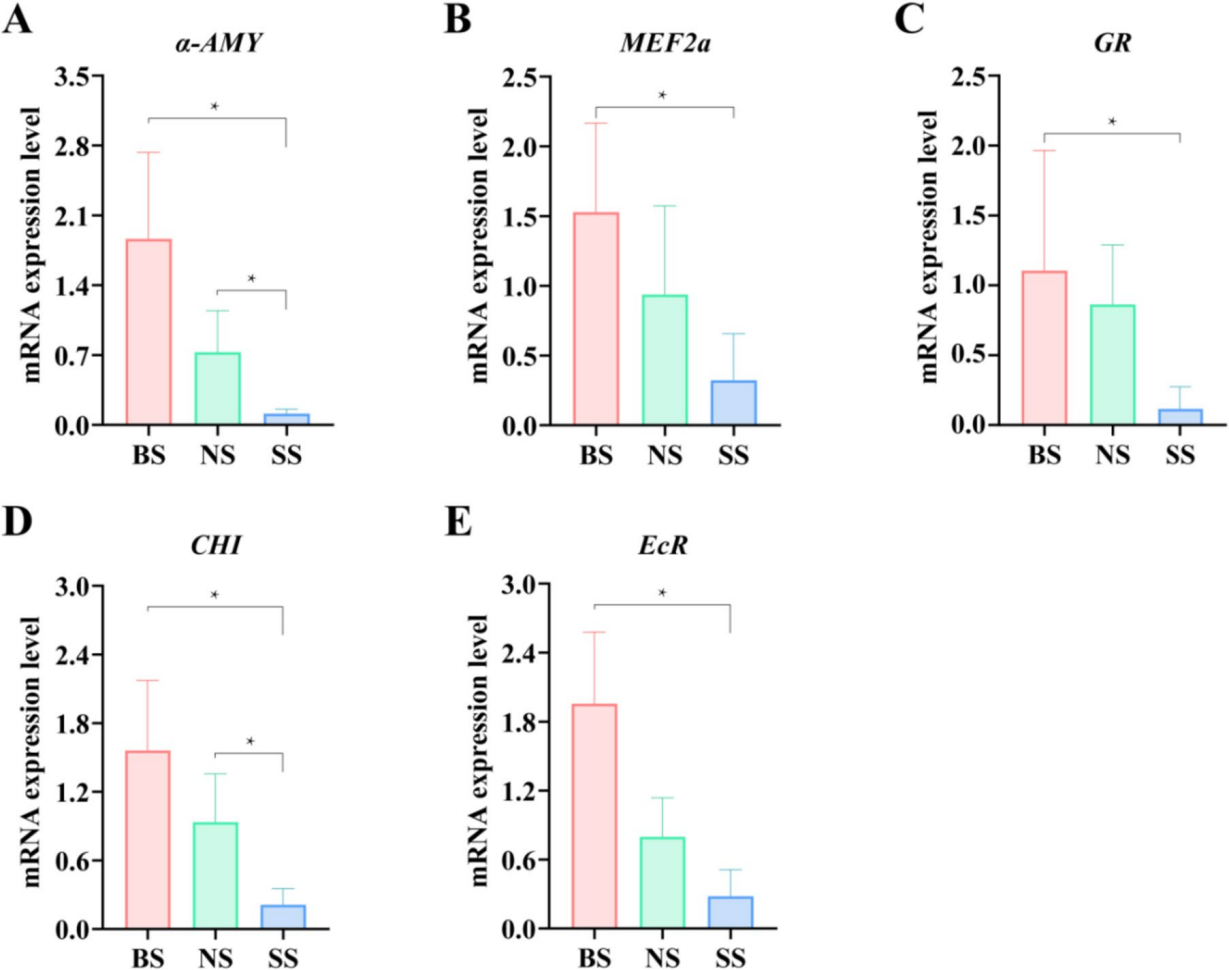
Histogram of expression levels of growth- and development-related genes in crayfish. **A**–**E**. The relative gene expression levels of alpha-amylase (*α-AMY*), myocyte-specific enhancer factor 2*a* (*MEF2a*), glutathione reductase (*GR*), chitinase (*CHI*), and ecdysone receptor (*EcR*). For each group consisted of three individual samples, all qRT-PCRs were performed in triplicate. Significance: *P* < 0.05 (*)

To further investigate the difference in gene expression levels in crayfish, five genes related to growth and development were selected by qRT-PCR (Fig. 6). The results showed a similar expression tendency as the high-throughput sequencing data and enzyme activity assays, despite some quantitative differences in expression levels. Specifically, the relative expression of *α-AMY*, *MEF2a*, *GR*, *CHI*, and *ECR* in BS was significantly higher than that in SS (*P* < 0.05), and that of *α-AMY* and *CHI* in NS was also significantly higher than that in SS (*P* < 0.05), while no significant difference was observed in others, suggesting that the growth and developmental regulatory levels of crayfish were affected by the composition of intestinal microbiota, which resulted in the final difference of sizes.

## Discussion

The intestine is considered a metabolic organ of hosts which is essential for growth and development (Cicala et al. 2020). The intestinal microbiota were found to be interrelated and to influence each other and become an important part of the host’s life, which forms a complex ecosystem with relative stability but dynamic changes (Zhang et al. 2020). The crayfish, as a benthic organisms, are greatly influenced by water and sediments. Their intestinal microbiota could be changed with the dominant status of bacteria in water and sediments, resulting in differences in digestion and absorption (Wang et al. 2020a). Therefore, we analyzed the intestinal microbiota characteristics of crayfish of three sizes in the same typical rice-crayfish coculture field by high-throughput sequencing technology combined with qRT-PCR and enzyme activity, investigating the relationship of water and sediments and the potential impact on digestion and absorption, which is valuable for managing and manipulating the intestinal microbiota of crayfish to achieve high productivity in practice.

The core microbiota are important for the physiological functions of the intestine, including the digestion and absorption of nutrients (Ghanbari et al. 2015; Hou et al. 2021; Wang et al. 2021). Previous studies have found that the dominant phyla of the intestinal microbiota mainly consist of Bacteroidota, Firmicutes, Proteobacteria, and Actinobacteria (Hou et al. 2021), and the relative abundance varied between the intestine, water, and sediments, a finding that agreed well with our results (Sun et al. 2023a,b; Wang et al. 2021). However, our results on the ratio of Firmicutes differed from those reported in previous studies. This discrepancy might be mainly attributed to external factors, such as environmental conditions and genetic background, why its intestinal bacterial flora might be changed. In general, Firmicutes and Bacteroidota are directly associated with energy metabolism (Gao et al. 2022; Semova et al. 2012). Among these, *Bacteroides* (De Filippo et al. 2010) and *Candidatus_Bacilloplasma* (Huang et al. 2018) play an important role in the growth of crayfish and are correlated with energy and lipid metabolism and weight variation (Gao et al. 2022; Semova et al. 2012). Meanwhile, *Bacteroides* also contributes to the degradation of polysaccharides from food, produces short-chain fatty acids, and obtains the necessary nutrients and energy needed for hosts (De Filippo et al. 2010), and *Candidatus_Bacilloplasma*, a bacterium widely occurring in the intestine of crayfish, could degrade recalcitrant C sources and become an intestinal mutualistic symbiont (Wang et al. 2004, 2020b). Combined with our results, *Bacteroides* was significantly higher in BS than in NS and SS, and *Candidatus_Bacilloplasma* gave similar results. In summary, these results indicated that the crayfish in BS had a better nutrient uptake and better growth performance, which is a valuable information for crayfish culture and provided a reference for probiotic addition.

Meanwhile, the variation in water and sediments might indirectly alter the intestinal microbiota of crayfish because of the difference in environmental tolerance (Nakashima et al. 2018). The intestinal microbiota should be more similar to the environment and might enter the intestine through the oral route (Soonthornchai et al. 2010). For this reason, the stability of the intestinal microbiota became especially important. When the intestinal bacterial community is changed, it could result in altered functional pathways, such as immune response and exogenous substance degradation, which could affect the growth of the host (Garibay-Valdez et al. 2020). Correlation networks have been suggested to be a powerful tool for exploring taxa coexistence in complex microbial communities (Jiang et al. 2019), which provides direct and valuable information on the microbial interactions between crayfish and their surroundings (Wang et al. 2021). In this study, a total of five phyla, *Chloroflexi*, Firmicutes, Actinobacteria, Bacteroidetes, and Proteobacteria, were shared. *Bacteroides* and *Candidatus_Bacilloplasma* corresponded to important nodes connecting the environment and intestine samples and were negatively correlated with the majority of bacteria in SS, which might cause the intestinal microbiota to be more susceptible to the influence of the external environment when the environment changes and influence the difference in growth and development in crayfish.

In addition, better digestion and absorption would benefit the growth of hosts (Gao et al. 2022). According to the available studies, we found that cellulolysis facilitated the digestion of eaten food, producing some beneficial metabolites, such as butyrate (Shin et al. 2015). More cellulolysis was not only an energy source for intestinal bacterial growth but also associated with the proliferation of intestinal cells (Lallès 2016). The development of intestinal stability could be promoted by cellulolysis, which is essential for improving growth, basically in agreement with the findings of this study. Moreover, in crustaceans, the expression levels of *α-AMY*, *MEF2a*, *GR*, *CHI*, and *EcR* were closely associated with digestion and absorption, molting, and muscle production (Gao et al. 2022; Zhang et al. 2019). Specifically, molting is a periodic and energetically costly event regulated by *EcR* and *CHI* (Cheng et al. 2023). *MEF2a* and *GR* were associated with muscle growth, while *α-AMY* was closely related to digestive and absorptive capacity and nutrient metabolism in crayfish (Zhang et al. 2021, 2023). In this study, we observed that all five growth-related genes were most highly expressed in BS. Combined with enzyme activity, the activity of digestive enzymes was significantly greater in BS than in NS and SS, which further validated the results of functional predictions. This result showed that there was a potential interaction between the intestinal microbiota of crayfish and the environmental microbiota, and more cellulolysis contributed to the growth and development of crayfish for better growth characteristics.

In conclusion, there were significant differences in the dominant intestinal microflora in crayfish between three sizes (BS, NS, and SS), including *Bacteroides* and *Candidatus_Bacilloplasma*, which were more abundant in BS, and where they could produce some beneficial metabolites through cellulolysis that could facilitate digestion and absorption. This could be one of the potential factors affecting the difference in crayfish sizes, which would be beneficial to assist farmers in improving the efficiency of culture.

## Supplementary Information

Below is the link to the electronic supplementary material.

Supplementary file1 (PDF 1107 KB)

## Data Availability

The datasets supporting the conclusions of the article were included within the article and its supplementary materials. The data presented in this study were deposited in the Sequence Read Archive (SRA) at the NCBI repository, accession number: PRJNA960913.
