# Supplementary material for: Microbiome analysis reveals the intestinal microbiota characteristics and potential impact of Procambarus clarkii
Source: Appl Microbiol Biotechnol. 2024 Jan 10;108:77. doi: 10.1007/s00253-023-12914-5 (PMC10781845; doi:10.1007/s00253-023-12914-5)

Supplemental Material

Journal:Applied Microbiology and Biotechnology

Microbiome analysis reveals the intestinal microbiota characteristics and potential impact of *Procambarus clarkii*

Ming Xu[1, #], Fulong Li[1, #], Xiaoli Zhang[2], Baipeng Chen[1], Yi Geng[1], Ping Ouyang[3], Defang Chen[1], Liangyu Li[2], Xiaoli Huang[1,*]

[1] Department of Aquaculture, College of Animal Science & Technology, Sichuan Agricultural University, Chengdu 611130, Sichuan, China.

[2] Fishery Research Institute, Chengdu Academy of Agriculture and Forestry Sciences, Wenjiang, Sichuan, China.

[3] Department of Basic Veterinary, College of Veterinary Medicine, Sichuan Agricultural University, Chengdu 611130, Sichuan, China

[#] These authors contributed equally to this work.

* Corresponding author:

Xiaoli Huang.

E-mail addresses: hxlscau@126.com.

Telephone: +86-181 8084 1626

Fax: no

Table S1

Sequencing characteristics

Sample type	Category	Replicate	Seq_num	Base_num	Mean_length	Min_length	Max_length
Intestine	BS	B1	41407	17566650	424.243485	262	464
		B2	44057	18637602	423.033843	202	477
		B3	42493	17982304	423.182736	345	489
	NS	N1	38093	16247787	426.529467	261	433
		N2	35791	15238193	425.754883	253	513
		N3	41400	17526121	423.336256	216	431
	SS	S1	39469	16823034	426.234108	255	477
		S2	43920	18703612	425.856375	265	445
		S3	38934	16601567	426.40281	261	431
Water	Water	W1	66533	27861962	418.769062	202	477
		W2	62061	26031939	419.457292	263	491
		W3	59857	25104598	419.409559	235	511
Sediment	Sediment	N1	65492	27295117	416.770247	203	528
		N2	65433	27248327	416.43096	203	491
		N3	63902	26793706	419.2937	203	493

Table S2

The current status of crayfish culture

**Data category**	**Index**	**Unit**	**Average**	**BS**	**NS**	**SS**
Breeding status	Full weight	g	20.05±4.78	25.98±1.08[a]	19.32±0.65[b]	14.84±1.39[c]
	Abdominal weight	g	4.73±0.94	5.57±0.35[a]	5.00±0.59[b]	3.62±0.36[c]
	Cephalothorax weight	g	10.98±2.68	14.08±1.41[a]	10.72±0.72[b]	8.13±0.95[c]
	Chela foot weight	g	4.34±1.71	6.33±1.05[a]	3.60±0.68[b]	3.09±1.05[b]
	Body length	cm	9.02±0.76	9.71±0.26[a]	9.01±0.61[b]	8.34±0.61[c]
	Cephalothorax length	cm	4.45±0.39	4.86±0.24[a]	4.35±0.23[b]	4.15±0.29[b]
	Abdominal length	cm	3.47±0.20	3.48±0.20[a]	3.52±0.12[a]	3.42±0.26[a]
	Cephalothorax width	cm	1.82±0.27	2.02±0.21[a]	1.83±0.18[a]	1.60±0.24[b]
	Abdominal width	cm	1.58±0.38	1.90±0.81[a]	1.61±0.36[b]	1.25±0.30[c]
	Cephalothorax height	cm	2.00±0.34	2.31±0.27[a]	2.01±0.28[b]	1.69±0.11[c]
	Abdominal height	cm	1.07±0.22	1.14±0.18[a]	1.15±0.21[a]	0.91±0.19[b]
	Sales price	¥ / 500g	26.7	32	26	22

[a], [b] and [c] represent $P < 0.05$, respectively.

Table S3

Water quality and weather data during sampling

Data category	Index	Unit	Average
Water quality	Water depth	m	2.23±0.05
	Water temperature	℃	27.07±0.38
	pH		8.13±0.05
	Dissolved oxygen	mg/L	7.13±0.06
	Ammonia nitrogen	mg/L	0.22±0.02
	Nitrite	mg/L	0.21±0.01
	Transparency	m	0.54±0.08
Weather date	Average temperature	℃	28.5
	Maximum temperature	℃	32.2
	Minimum temperature	℃	25.5
	Humidity	%	76
	Wind speed	m/s	1.3
	Daily precipitation	mm	0
	Atmospheric pressure	hpa	956

Table S4

Diversity of microbial communities

Sample type	Category	Replicate	Sobs	Shannon	Simpson	Ace	Chao	Coverage
Intestine	BS	B1	394	3.09191	0.121863	458.622772	476.269231	0.99765
		B2	305	2.889928	0.135741	384.522481	375.895833	0.998023
		B3	335	3.54593	0.215705	419.285548	445.157895	0.997778
	NS	N1	327	3.046793	0.097813	526.794816	520.642857	0.996888
		N2	362	2.888349	0.121748	414.103623	432.222222	0.997701
		N3	236	2.876829	0.106824	289.210522	282.941176	0.998581
	SS	S1	299	2.937398	0.104244	351.579736	347.065217	0.998255
		S2	281	2.665294	0.146217	377.324717	364.068182	0.997984
		S3	283	2.354894	0.194787	332.73549	322.722222	0.99826
Water	Water	W1	1245	5.093688	0.014956	1760.586816	1790.740741	0.989591
		W2	1354	5.271772	0.012478	2218.62114	1974.155556	0.988444
		W3	1326	5.213053	0.012178	1860.289945	1874.513514	0.988982
Sediment	Sediment	N1	2733	6.624547	0.00444	3285.60213	3291.923077	0.978657
		N2	2852	6.741212	0.003251	3363.015448	3298.892	0.979684
		N3	2602	6.245201	0.010322	3193.654731	3174.36	0.979288

Table S5

Network node attributes and center coefficients

Node _ID	Node_Name	Degree	Weighted degree	Degree Centrality	Closeness Centrality	Betweenness Centrality
1	BS	/	/	0.378378378	0.46835443	0.038206506
2	NS	/	/	0.405405405	0.480519481	0.047282611
3	SS	/	/	0.324324324	0.445783133	0.024168997
4	Water	/	/	0.837837838	0.822222222	0.444021062
5	Sediment	/	/	0.756756757	0.725490196	0.35172623
6	*Acidobacteriota*	2	4099	0.108108108	0.521126761	0.006098296
7	*Actinobacteriota*	5	18542	0.135135135	0.536231884	0.009859382
9	*Bacteroidota*	5	69353	0.135135135	0.536231884	0.009859382
10	*p_Bdellovibrionota*	2	279	0.054054054	0.493333333	0.000752742
11	*p_Caldisericota*	2	150	0.054054054	0.493333333	0.000752742
12	*Campilobacterota*	2	238	0.054054054	0.493333333	0.000752742
13	*Chloroflexi*	5	11880	0.135135135	0.536231884	0.009859382
14	*Cyanobacteria*	5	1346	0.135135135	0.536231884	0.009859382
16	*Deinococcota*	4	124	0.108108108	0.493333333	0.004402631
17	*Desulfobacterota*	5	7221	0.135135135	0.536231884	0.009859382
18	*Firmicutes*	5	141156	0.135135135	0.536231884	0.009859382
19	*Gemmatimonadota*	2	308	0.054054054	0.493333333	0.000752742
20	*p_Latescibacterota*	2	257	0.054054054	0.493333333	0.000752742
21	*MBNT15*	2	426	0.054054054	0.493333333	0.000752742
22	*Margulisbacteria*	1	88	0.027027027	0.456790123	0
23	*Methylomirabilota*	2	218	0.054054054	0.493333333	0.000752742
24	*Myxococcota*	3	2200	0.081081081	0.506849315	0.003182375
26	*Nitrospinota*	2	199	0.054054054	0.493333333	0.000752742
27	*Nitrospirota*	3	1934	0.081081081	0.506849315	0.003436599
28	*Patescibacteria*	4	214	0.108108108	0.493333333	0.004402631
29	*Planctomycetota*	5	1064	0.135135135	0.536231884	0.009859382
30	*Proteobacteria*	5	102343	0.135135135	0.536231884	0.009859382
31	*RCP2-54*	2	139	0.054054054	0.493333333	0.000752742
33	*Spirochaetota*	2	425	0.054054054	0.493333333	0.000752742
34	*Sva0485*	3	928	0.081081081	0.506849315	0.003182375
35	*TA06*	2	77	0.054054054	0.493333333	0.000752742
36	*Verrucomicrobiota*	5	2787	0.135135135	0.536231884	0.009859382
37	*Zixibacteria*	1	73	0.027027027	0.425287356	0
38	*Bacteria*	4	166	0.108108108	0.493333333	0.004402631

Table S6

Network node attributes and center coefficients of correlation network in BS.

Node_name	Degree	Clustering	Degree_Centrality	Closeness_Centrality	Betweenness_Centrality
g__Citrobacter	17	0.78676	0.58621	0.65909	0.01371
Acinetobacter_johnsonii	7	1	0.24138	0.48333	0
Acinetobacter	11	0.89091	0.37931	0.56863	0.00291
Dysgonomonas	20	0.72105	0.68966	0.76316	0.04538
Anaerorhabdus_furcosa_group	20	0.81579	0.68966	0.76316	0.01994
ZOR0006	22	0.77489	0.75862	0.80556	0.02118
RsaHf231	22	0.77489	0.75862	0.80556	0.02118
Bacteroides	22	0.77489	0.75862	0.80556	0.02118
Weeksellaceae	22	0.77489	0.75862	0.80556	0.02118
Candidatus_Bacilloplasma	23	0.73913	0.7931	0.82857	0.03197
g__Vibrio	24	0.7029	0.82759	0.85294	0.04754
Empedobacter	24	0.7029	0.82759	0.85294	0.04754
Defluviicoccus_sp.	9	0.94444	0.31034	0.54717	0.00121
Methylocystis	18	0.84967	0.62069	0.725	0.00904
Dechloromonas	13	0.94872	0.44828	0.59184	0.00109
Xanthobacteraceae	16	0.80833	0.55172	0.63043	0.01035
Steroidobacteraceae	16	0.80833	0.55172	0.63043	0.01035
Anaerolineaceae	16	0.8	0.55172	0.64444	0.01176
Clostridiaceae	18	0.87582	0.62069	0.70732	0.00868
Incertae_Sedis	21	0.71429	0.72414	0.78378	0.09674
Novosphingobium	19	0.84795	0.65517	0.74359	0.01335
Mycobacterium	19	0.84795	0.65517	0.74359	0.01335
Ramlibacter	18	0.87582	0.62069	0.70732	0.00868
g__hgcI_clade	18	0.87582	0.62069	0.70732	0.00868
Hydrogenophaga	17	0.88971	0.58621	0.69048	0.00739
Limnohabitans_sp._103DPR2	11	0.85455	0.37931	0.54717	0.00513
Limnohabitans	18	0.81046	0.62069	0.70732	0.028
env.OPS_17	11	0.74545	0.37931	0.52727	0.01273
Comamonadaceae	3	1	0.10345	0.46032	0
g__C39	5	0.8	0.17241	0.47541	0.00164

Table S7

Network node attributes and center coefficients of correlation network in NS.

Node_name	Degree	Clustering	Degree_Centrality	Closeness_Centrality	Betweenness_Centrality
Clostridiaceae	10	0.95556	0.34483	0.46774	0.00062
Mycobacterium	9	0.83333	0.31034	0.46032	0.01067
Limnohabitans	18	0.66667	0.62069	0.64444	0.05959
g__hgcI_clade	17	0.73529	0.58621	0.63043	0.0346
Ramlibacter	17	0.73529	0.58621	0.63043	0.0346
Mycobacterium	18	0.69935	0.62069	0.64444	0.05097
Incertae_Sedis	19	0.65497	0.65517	0.65909	0.06885
Hydrogenophaga	13	0.79487	0.44828	0.58	0.01211
f__env.OPS_17	13	0.75641	0.44828	0.49153	0.02425
g__hgcI_clade	12	0.80303	0.41379	0.48333	0.02177
sp._103DPR2	12	0.84848	0.41379	0.48333	0.00373
Bacteroides	17	0.75735	0.58621	0.67442	0.04424
ZOR0006	17	0.75735	0.58621	0.67442	0.04424
Empedobacter	17	0.75735	0.58621	0.67442	0.04424
RsaHf231	18	0.71242	0.62069	0.69048	0.07381
Weeksellaceae	17	0.75735	0.58621	0.67442	0.04424
Candidatus_Bacilloplasma	17	0.75735	0.58621	0.67442	0.04424
Exiguobacterium	14	0.68132	0.48276	0.55769	0.06729
Citrobacter	14	0.68132	0.48276	0.55769	0.06729
Novosphingobium	17	0.71324	0.58621	0.63043	0.04343
Methylocystis	14	0.83516	0.48276	0.63043	0.02212
Dechloromonas	11	0.89091	0.37931	0.52727	0.01878
Steroidobacteraceae	13	0.76923	0.44828	0.54717	0.03773
Anaerolineaceae	12	0.78788	0.41379	0.53704	0.03223
Acinetobacter	7	0.57143	0.24138	0.39726	0.07143
sp._DY2632	1	0	0.03448	0.28713	0
Xanthobacteraceae	3	1	0.10345	0.37662	0
Defluviicoccus_sp.	4	1	0.13793	0.37662	0
Comamonadaceae	4	0.66667	0.13793	0.34524	0.00076
g__C39	7	0.7619	0.24138	0.44615	0.00985

Table S8

Network node attributes and center coefficients of correlation network in SS.

Node_name	Degree	Clustering	Degree_Centrality	Closeness_Centrality	Betweenness_Centrality
Citrobacter	14	0.73626	0.48276	0.58	0.01143
Acinetobacter	7	0.95238	0.24138	0.46774	0.00031
g__Vibrio	8	0.71429	0.27586	0.54717	0.00683
Anaerorhabdus_furcosa_group	19	0.64327	0.65517	0.74359	0.07753
Weeksellaceae	17	0.74265	0.58621	0.67442	0.02117
Candidatus_Bacilloplasma	17	0.74265	0.58621	0.67442	0.02117
RsaHf231	17	0.74265	0.58621	0.67442	0.02117
ZOR0006	19	0.66082	0.65517	0.70732	0.04413
Bacteroides	20	0.62632	0.68966	0.725	0.07764
Defluviicoccus_sp.	8	0.82143	0.27586	0.47541	0.00228
Xanthobacteraceae	13	0.79487	0.44828	0.56863	0.00796
Steroidobacteraceae	12	0.81818	0.41379	0.50877	0.00481
Anaerolineaceae	12	0.81818	0.41379	0.55769	0.00627
Methylocystis	13	0.83333	0.44828	0.59184	0.00728
Dechloromonas	12	0.75758	0.41379	0.59184	0.0118
Novosphingobium	17	0.71324	0.58621	0.70732	0.03151
Incertae_Sedis	19	0.61988	0.65517	0.74359	0.08526
Limnohabitans	18	0.64706	0.62069	0.725	0.07981
g__hgcI_clade	16	0.775	0.55172	0.69048	0.0211
Mycobacterium	17	0.73529	0.58621	0.70732	0.03094
Ramlibacter	17	0.73529	0.58621	0.70732	0.03094
Hydrogenophaga	13	0.75641	0.44828	0.60417	0.0315
g__hgcI_clade	12	0.78788	0.41379	0.59184	0.03528
sp._103DPR2	13	0.75641	0.44828	0.60417	0.0147
Comamonadaceae	6	0.66667	0.2069	0.42029	0.00345
Rhodoferax	6	0.66667	0.2069	0.47541	0.00565
Polynucleobacter_asymbioticus	4	0.66667	0.13793	0.40845	0.00123
Mycobacterium	10	0.8	0.34483	0.53704	0.01049
bacterium_g_C39	8	0.78571	0.27586	0.50877	0.00665
env.OPS_17	14	0.63736	0.48276	0.58	0.04588

Fig. S1 Species annotation and assessment.

A. Rank-abundance curve of samples. The x-axis represents the ranking level of the number of OTUs at the taxonomic level, and the y-axis represents the relative percentage of the number of species. B. Dilution curves of samples at OTU levels.

A

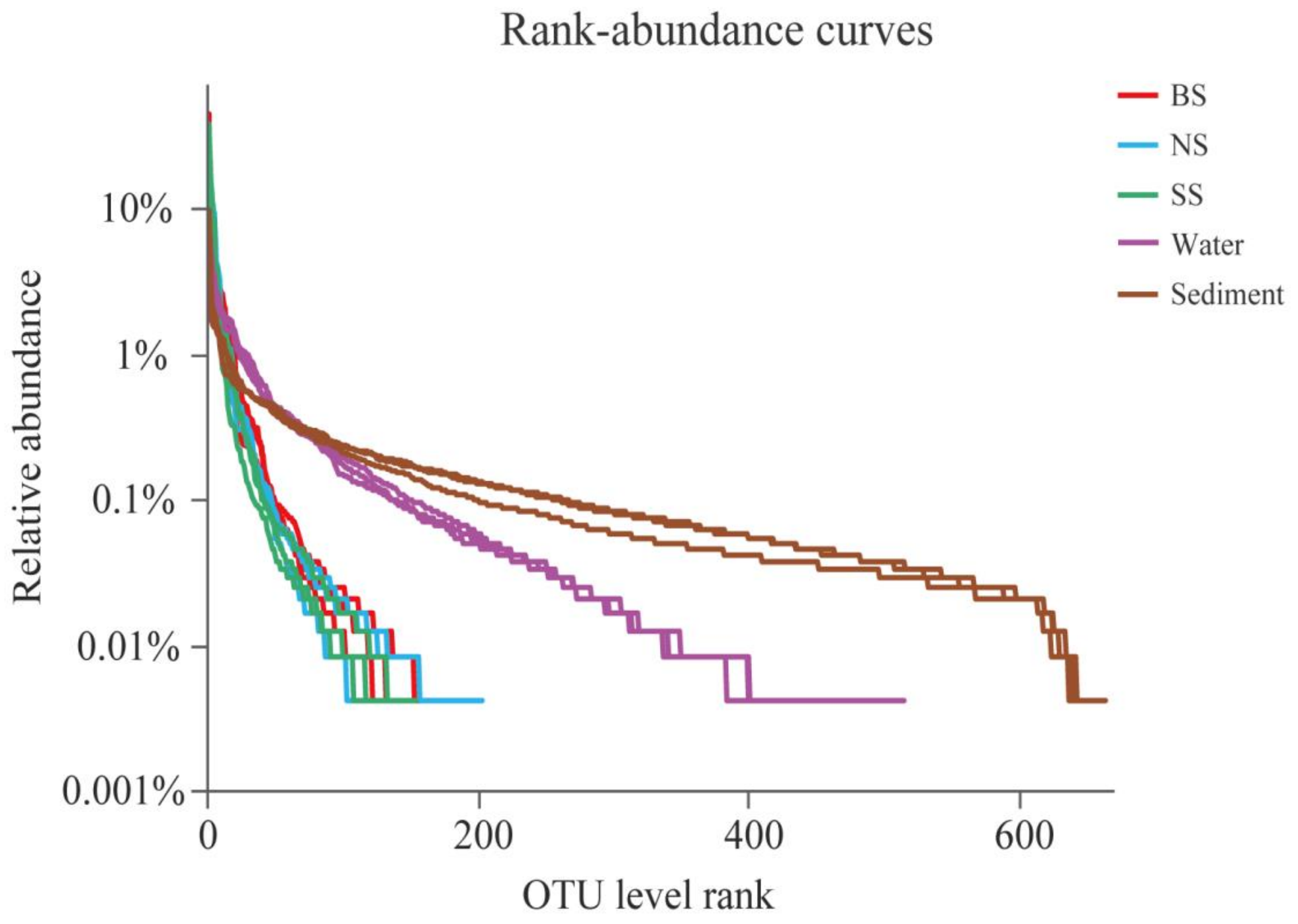

B

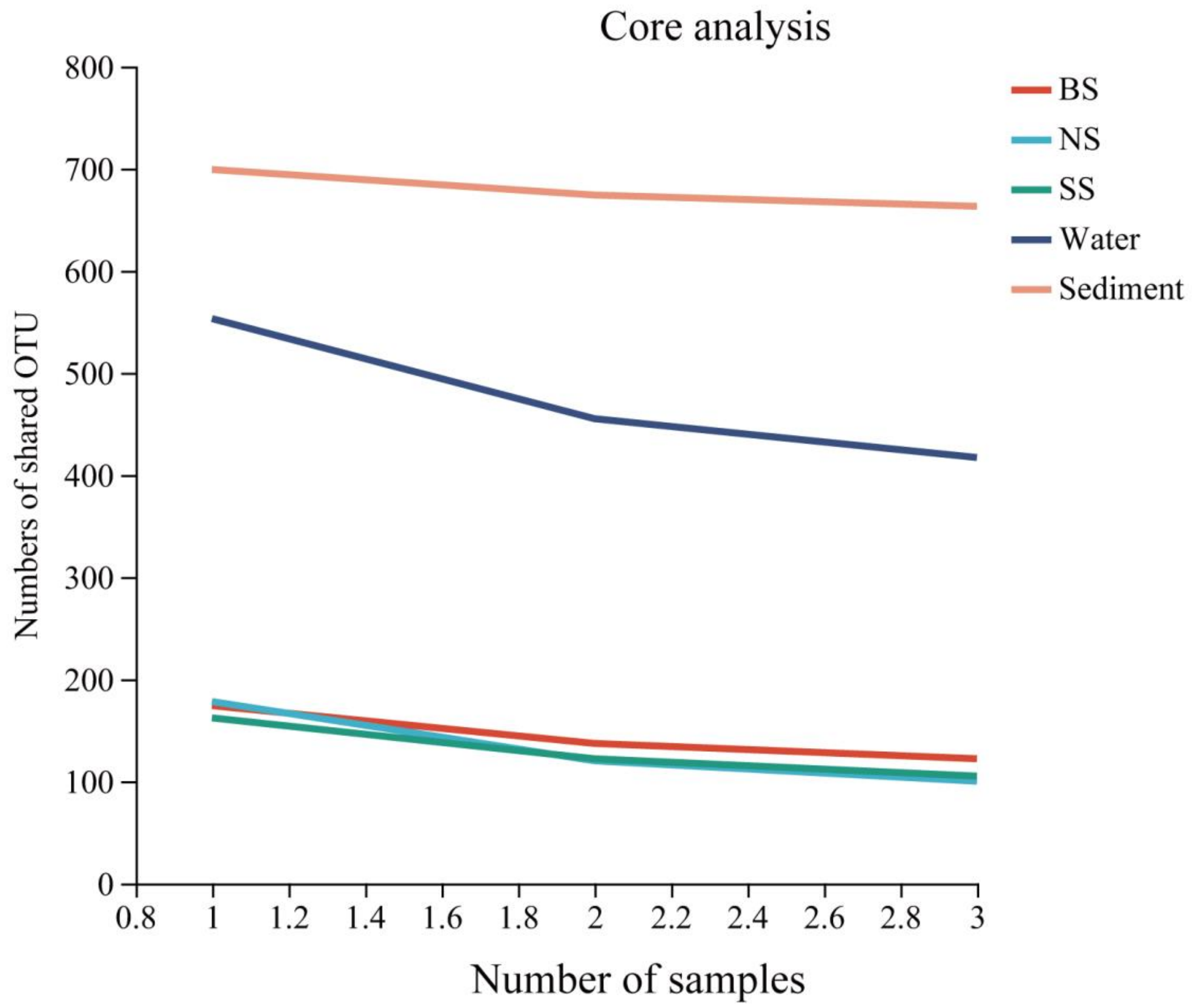

Fig. S2 Annotation and evaluation of species.

A. Venn diagram of the number of bacterial species annotated at the OTU level for different types of samples and shared bacteria in center. B. Principal component analysis (PCA) of bacterial communities in different groups (R = 0.9970, *P* = 0.001000).

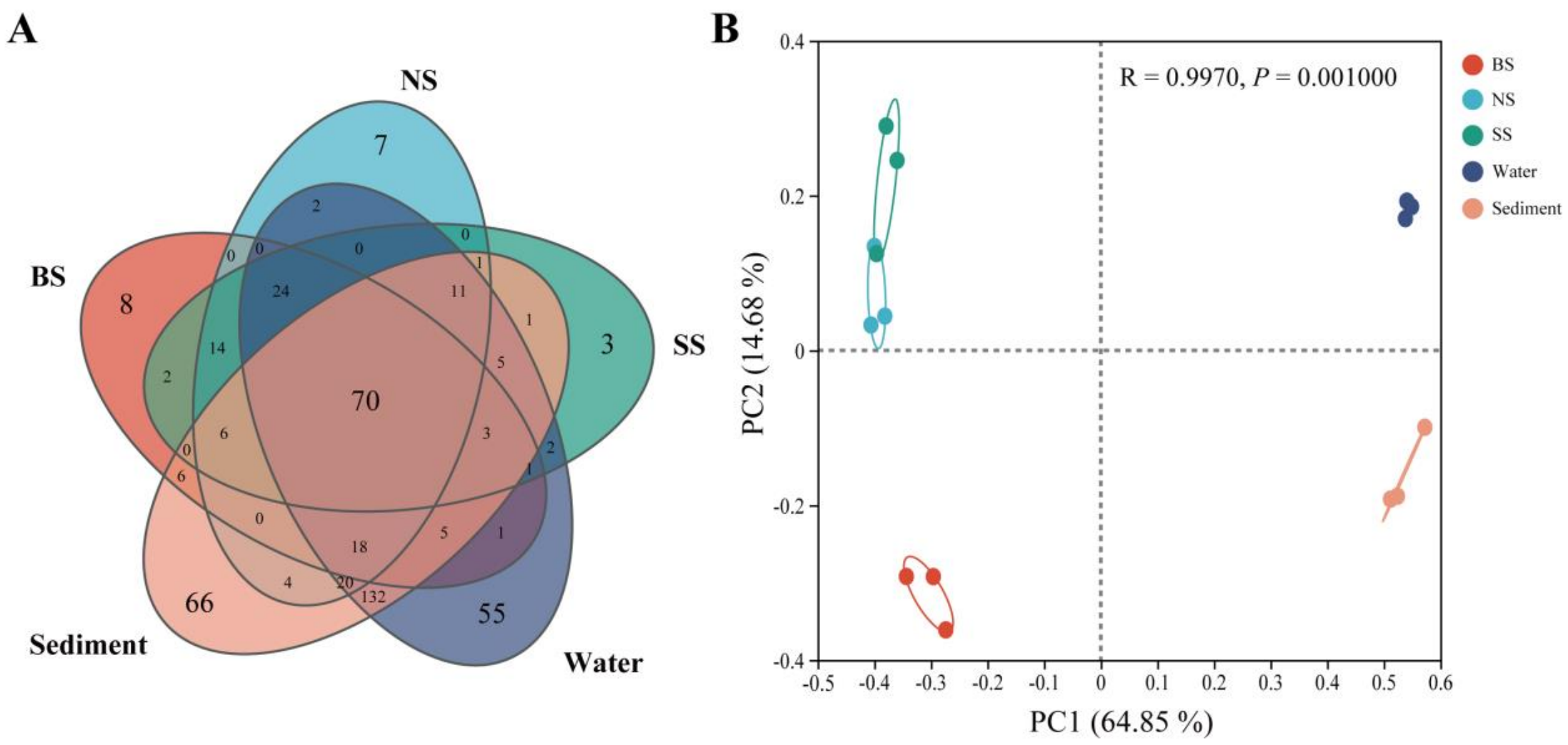

Fig. S3 Circos sample-genus relationship map.

Visualization reflects the proportion of dominant genus distribution in intestine sample, and the proportion of each dominant genus distribution in different samples.

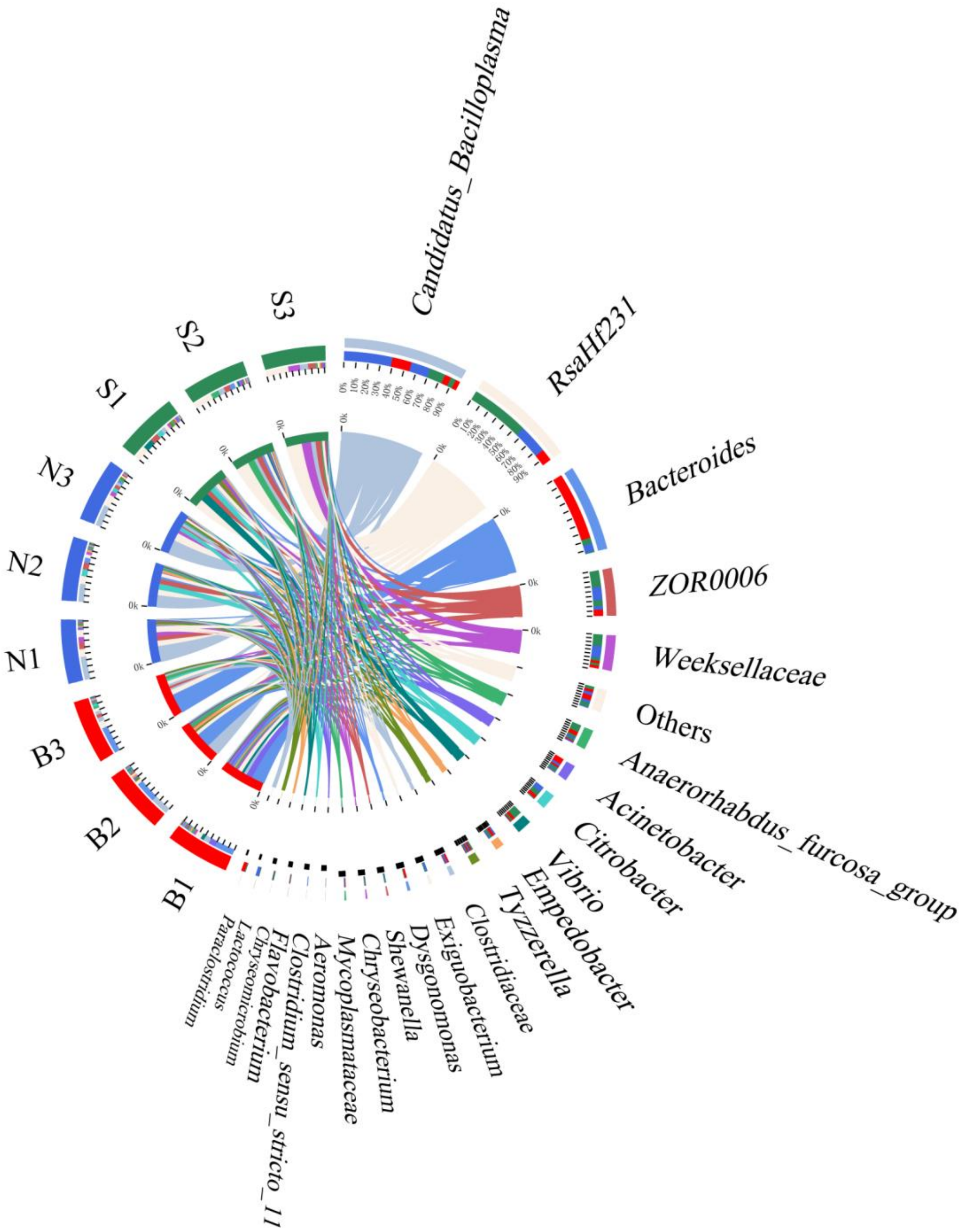

Fig. S4 Structure of the bacterial community in water and sediments.

Pie charts, annotated at the phylum level for water (A) and sediment (B) samples with bacterial communities.

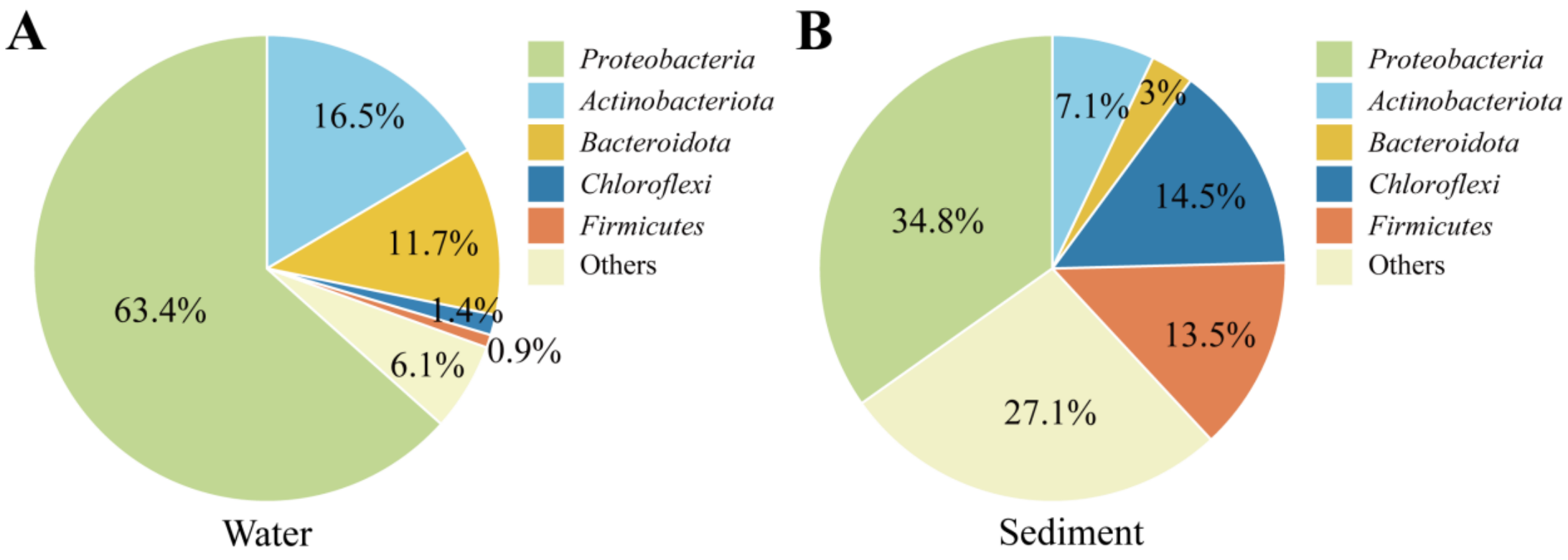

Fig. S5 Hierarchical clustering analysis of taxa in collected samples of different groups.

The UPGMA algorithm to visualize the degree of variation in community composition in different samples. The length between branches represents the distance between samples, and different groups are presented in different colors. The right side of the cluster tree shows the composition of the dominant species in each sample.

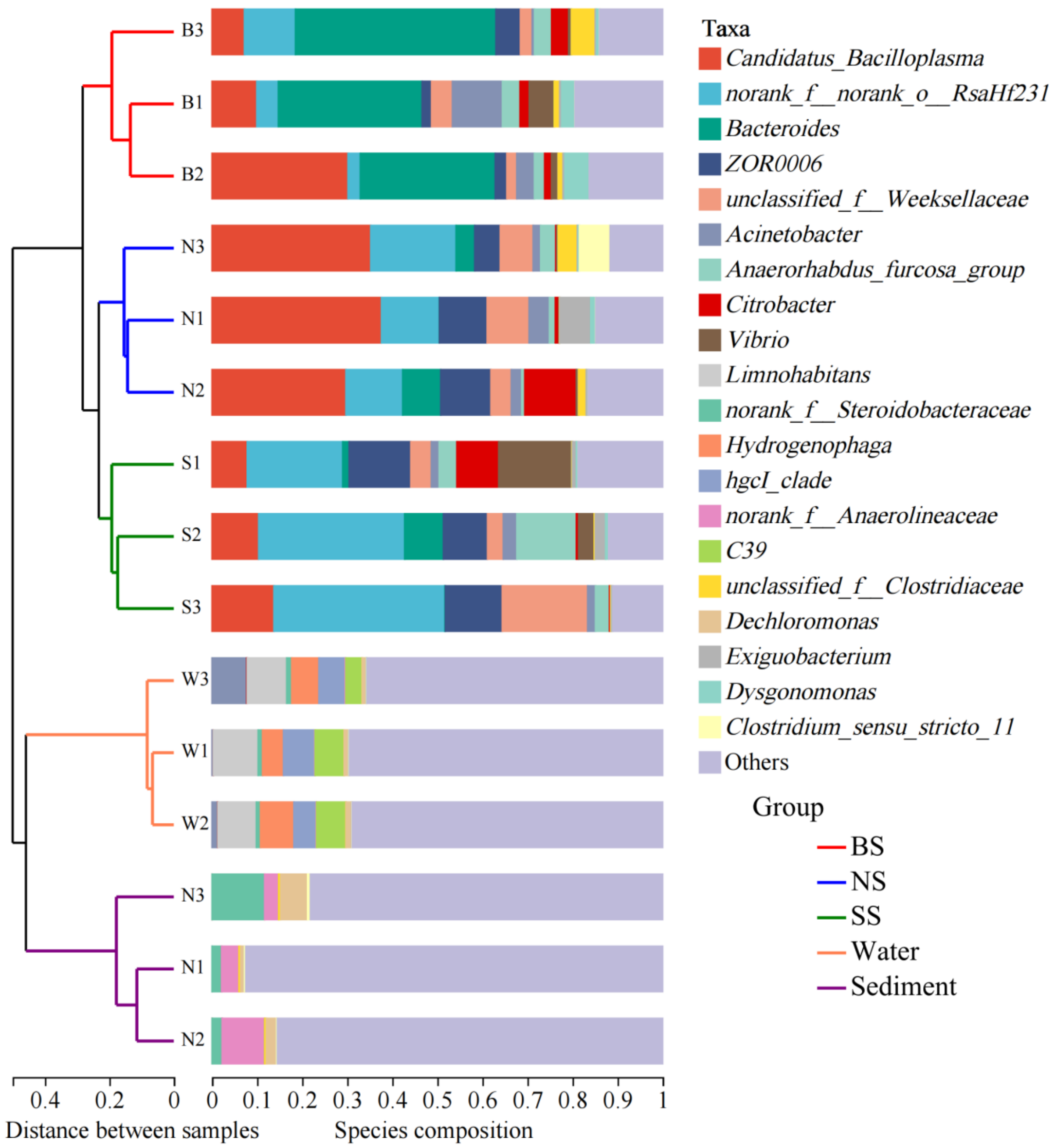

Fig. S6 Correlation analysis between the taxa of the intestine, water, and sediments.

The connecting line between the sample node and the species node represents the inclusion of the species in the sample, showing the species with abundance greater than 30.

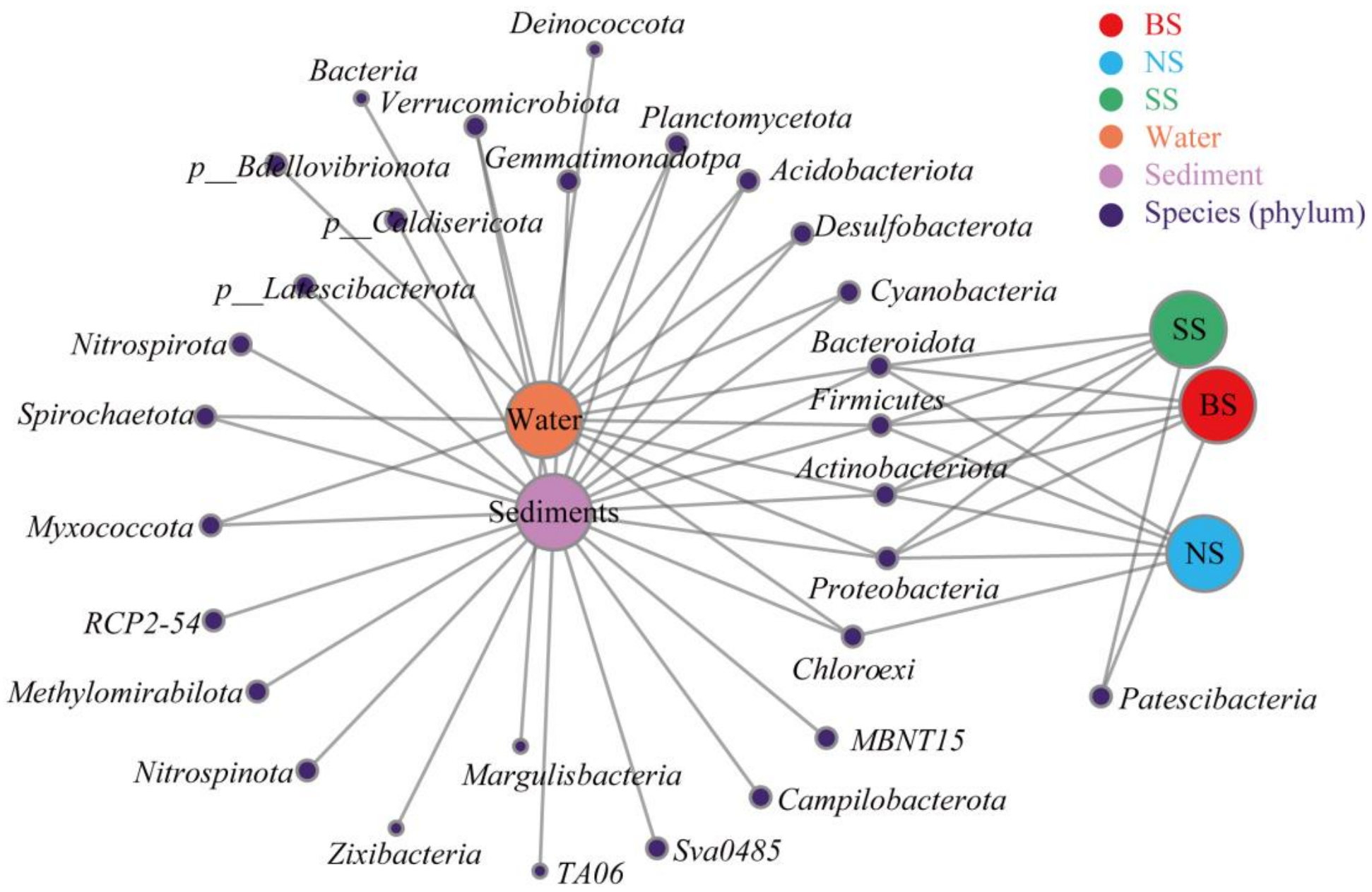

Supplement: Supplementary file 1 [file 253_2023_12914_MOESM1_ESM.pdf]
